# Trap Comparison for Surveillance of the Western Tree Hole Mosquito, *Aedes sierrensis* (Diptera: Culicidae)

**DOI:** 10.1093/jisesa/iez131

**Published:** 2020-01-09

**Authors:** Luis Fernando Chaves, Nadja Reissen, Gregory S White, Scott Gordon, Ary Faraji

**Affiliations:** 1 Instituto Costarricense de Investigación y Enseñanza en Nutrición y Salud (INCIENSA), Tres Rios, Cartago, Apartado, Costa Rica; 2 Salt Lake City Mosquito Abatement District, Salt Lake City, UT; 3 Biogents AG, Regensburg, Germany

**Keywords:** mosquito surveillance, trap efficacy, *Culex pipiens*, *Culex tarsalis*, *Culiseta incidens*

## Abstract

The western tree hole mosquito, *Aedes sierrensis* (Ludlow), is a common nuisance mosquito and vector of *Dirofilaria immitis* (Leidy), the etiologic agent of dog heartworm, in western North America. Here, we compare weekly mosquito collections made with Mosquito Magnet (MM) traps, Biogents Sentinel (BGS) traps, and Biogents Bowl (BGS Bowl) traps set in Salt Lake City, UT, from the start of June to mid-August 2017. We found the number of mosquitoes decreased with rainfall and temperature independently of trap type. The highest number of mosquitoes were caught by BGS traps baited with carbon dioxide (CO_2_) and BG lure, which collected 62% (*n* = 422) of all mosquitoes, followed by the MM at 31% (*n* = 213), and both the BGS and BG Bowl with BG lure had 3.5% (*n* = 24) each. *Aedes sierrensis* females were caught weekly at similar densities (mean ± SD) in BGS with CO_2_ and lure (1.17 ± 2.93) and the MM (1.17 ± 2.66) traps during the study period. Given that BGS with CO_2_ and lure traps have several operational advantages over MM traps, including a quicker setup, smaller size, and lower cost, we consider BGS with CO_2_ and lure traps as the best suited surveillance tool to detect and remove *Ae. sierrensis* in the western United States and similar settings throughout North America.

The western tree hole mosquito, *Aedes sierrensis* (Ludlow), is a common mosquito species inhabiting natural tree holes within oak and mixed deciduous forests, near rural and suburban environments in western North America (Darsie and Ward 2005, Thiemann et al. 2017). The species is distributed from southern California to British Columbia in the north, and appears to reach its eastern distribution in the high desert state of Utah (Darsie and Ward 2005, Farajollahi and Price 2013). *Aedes sierrensis* may also occasionally colonize artificial containers with high levels of organic debris, such as leaf litter (Farajollahi and Price 2013). From a veterinary perspective, *Ae. sierrensis* is a major vector of *Dirofilaria immitis*, a parasitic nematode causing heartworm disease in dogs, as shown by several studies in the western United States (Walters and Lavoipierre 1982, Scoles et al. 1993, Scoles and Kambhampati 1995). *Aedes sierrensis* is also a major pest which readily bites humans and other mammals; however, its role as a medically important vector is limited (Bohart and Washino 1978). For example, *Ae. sierrensis* is known to have a low vectorial capacity to transmit West Nile virus (WNV), despite being vectorially competent for that virus, as inferred from laboratory studies (Goddard et al. 2002). *Aedes sierrensis* is also an unlikely Northway virus vector (Kramer et al. 1993). Host preference in *Ae. sierrensis* is primarily mammalophilic, with a high preference toward humans and dogs in peridomestic habitats (Egerter and Anderson 1989), but the mosquito will also readily feed on wild mammals if they are locally abundant; while occasional avian blood meals have also been detected from this species (Thiemann et al. 2017). The strong feeding preference toward hosts found in large abundance is not surprising, given the fact that *Ae. sierrensis* is a weak flyer and does not disperse far from its larval habitat (Kline 2007).

Several studies have also investigated adult *Ae. sierrensis* population ecology. For example, using human landing catches it has been shown that presence and movement is favored in high canopy cover habitats found in deciduous forests (Bennett 1980). *Aedes sierrensis* adults have also been collected using aspirators and several trap types, including modified Magoon traps baited with live rabbits and carbon dioxide (CO_2_; Garcia et al. 1988), CO_2_-baited Fay-Prince traps (Garcia et al. 1989), and duplex cone traps (Washburn et al. 1992). From these studies, CO_2_-baited Fay-Prince traps were reported to have the best efficacy, by capturing the highest number of mosquitoes, which was also linearly correlated with human landing catches (Garcia et al. 1989, Washburn et al. 1992). CO_2_-baited Fay-Prince traps and ovitraps were used in a 3-year longitudinal study, within dense oak woodlands in the Coast Range of northern California, showing that adult *Ae. sierrensis* activity persisted longer into the season in areas with dense canopy cover, and that *Ae. sierrensis* abundance was correlated with air temperature, not rainfall (Woodward et al. 2003). More recently, Thiemann et al. (2017) conducted a study using CO_2_-baited CDC style traps and aspirators inside walk in resting boxes to collect *Ae. sierrensis* and other mosquito species, finding that adult *Ae. sierrensis* abundance peaked at the start of the mosquito season in April and May annually. These unimodal peaks likely emerge from the univoltine biology of *Ae. sierrensis*, where adults emerge from overwintering larvae (Hawley 1985); however, additional broods may be possible during years of favorable environmental conditions with excessive rainfall and warmer temperatures (Carpenter and LaCasse 1955).

Despite valuable information about the ecology of *Ae. sierrensis*, little is known about traps that could serve for both its surveillance and removal on a routine basis. The Salt Lake City Mosquito Abatement District (SLCMAD) is one of the few mosquito programs in the United States that deploys an active tree hole control program. This program has been primarily developed in response to nuisance service requests caused by local populations of *Ae. sierrensis.* The program relies on inspection and application of residual larvicide products in tree holes using two teams comprised of two mosquito inspectors on each team. Within the jurisdiction of SLCMAD, more than 3,500 tree holes have been geolocated and are annually inspected/treated; with new locations added through additional field surveillance every season. Residential service requests provide excellent opportunities to detect new tree holes, as many of the uninspected trees are located in private backyards. Since adult *Ae. sierrensis* in Utah are primarily restricted to residential habitats within a close vicinity to their larval development sites, SLCMAD has utilized adult removal trapping as a viable control option (Kline 2007). Mosquito Magnet (MM) traps have been used effectively for both surveillance and removal of adult *Ae. sierrensis* within Salt Lake City for many years (Houggard and Dickson 1999). However, MM traps are expensive and difficult to deploy in the field because of their size and excessive weight. It has also been difficult to acquire replacement parts and components for the older MM trap types. As a result, SLCMAD has been investigating newer trap types that may be utilized in place of MMs when conducting surveillance and removal trapping of local populations of *Ae. sierrensis.*

An alternative to the MMs would be a trap that is both more affordable and easier to transport to various locations. A potential new surveillance option may be the Biogents Sentinel (BGS) traps, which have become the gold standard for collection of container-inhabiting *Aedes* species (Farajollahi et al. 2009, Chaves et al. 2013, Rochlin et al. 2015, Ng et al. 2018). These traps are capable of removing as many mosquitoes as MM and other trap types (Lühken et al. 2014), while also being potentially useful to mosquito control programs looking to develop an active surveillance program for endemic and invasive mosquito species. Here, we present the results of a field trial designed to compare MM traps with baited BGS and Biogents Bowl traps. We compare how these traps, with different mosquito attractants, were able to collect *Ae. sierrensis* and other common peridomestic mosquitoes at suburban locations within Salt Lake City, UT. We report on the efficacy of these trap traps to collect mosquitoes and the relationship between mosquito abundance and environmental variables.

## Materials and Methods

### Study Site and Mosquito Collections

**Fig. 1.**
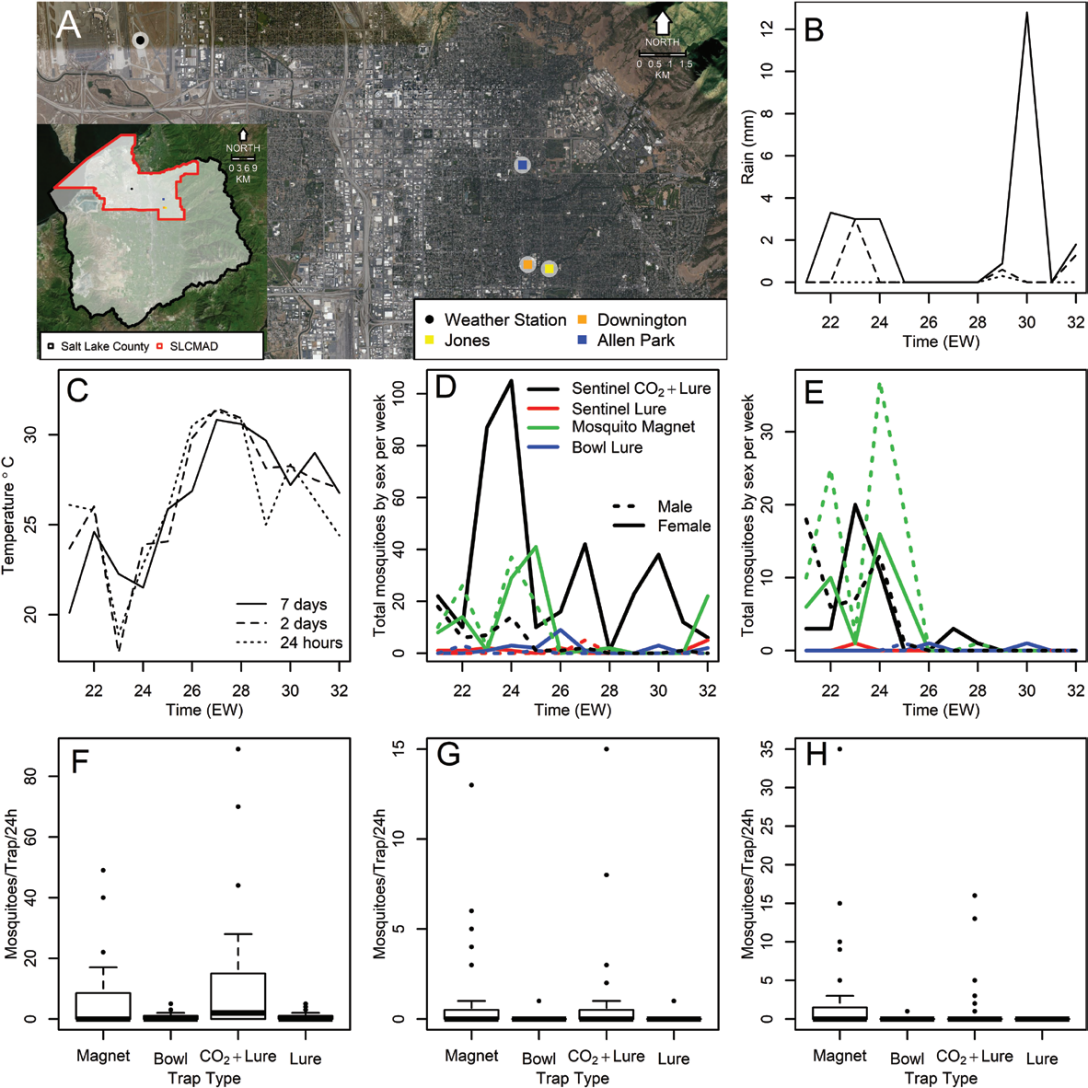
Study site, time series, and boxplots. (A) Weather station and sampling location map. The inset map highlights Salt Lake City and Salt Lake County, UT, and the area containing the three sampling locations and the weather station, for further details please refer to the inset legend. Locations are color coded as indicated in the inset legend of the main map. The maps were made using Google images as base. (B) Weekly rainfall, the inset legend indicates the line type associated with each of the three time scales we considered. (C) Weekly temperature, the line type indicates the time scale, see inset legend for B for details. (D) Weekly mosquito abundance for all species, color indicates trap type and sex is represented by the line dashing. For further details please refer to the inset legend. (E) Weekly *Aedes sierrensis* adult mosquito abundance, color indicates trap type and sex is represented by dashed line. For further details please refer to the inset legend of D. In B, C, D, and E, the temporal scale is presented in CDC MMWR epidemiological weeks (EW). (F) Boxplot of mosquito abundance for all species by trap type and bait combination (G) Boxplot of adult female *Ae. sierrensis* abundance by trap type and bait combination. (H) Boxplot of adult male *Ae. sierrensis* abundance by trap type and bait combination. In all boxplots, presented in E, G, and H, lines indicate the median of the distribution.

We selected three sampling locations in wooded areas of suburban Salt Lake City (40°45′0″N, 111°52′58.8″W; Fig. 1A). At each sampling location, we deployed four traps, including one MM trap (Mosquito Magnet Independence model, Woodstream Corp., Littiz, PA) operated by burning gas from a propane tank which generates CO_2_ and heat; one Biogents Bowl trap (BG Bowl, Biogents Sentinel, Regensburg, Germany) baited with a human skin scent (BG lure or lure hereafter); and two BGS traps (Biogents Sentinel 2) baited with BG lure. One of the BGS (CO_2_ and lure) traps was also supplemented with CO_2_ using a 20 oz paintball style CO_2_ tank (Tippmann Sports, Fort Wayne, IN) with a regulator calibrated to release CO_2_ at a rate of 220 ml/min. These traps were placed at four fixed sites within each of the three sampling locations. Within each site, traps were placed 10 m from each other, and were rotated to avoid systematic bias in mosquito collections. The distance between traps was chosen to ensure traps were collecting samples from the same mosquito community. From 1 June 2017 (CDC MMWR week 21) to 15 August 2017 (CDC MMWR week 32), traps were simultaneously placed at each study location and operated for 24 h starting at 8:00 a.m. At each location, traps were uniformly set so that collection openings were at approximately at 0.5 m height. Shortly after finishing trap operation, mosquitoes were removed and killed by freezing before enumeration and identification using the taxonomic key by Darsie and Ward (2005).

### Weather Data

To quantify the impacts of weather on mosquito collections, we downloaded daily rainfall and average temperature data from the Salt Lake City airport weather station (Station code: USW00024127) using the KNMI climate explorer available at http://climexp.knmi.nl/start.cgi. We then generated six weather time series, three for cumulative rainfall and three for average daily temperature, comprising data for: 1) the day traps were removed, 2) the days when traps were set and removed, and 3) the 7 d ending the day traps were removed. These time series were generated to account for different temporal scales at which weather variability might impact mosquito collections. Rainfall (Fig. 1B) only occurred on the day (24 h) traps were removed during the ninth week of sampling, while it also occurred while traps were collecting mosquitoes during the third and twelfth week of sampling (2 d). One week (7 d) cumulative rainfall occurred from the second to the fourth, ninth to tenth, and on the twelfth sampling week (Fig. 1B). Meanwhile, temperature fluctuated between 18 and 32°C, being colder at the start of the study; fluctuations were smaller for the 7-d estimates (Fig. 1C).

### Statistical Analysis

To select the appropriate time scale at which weather variability impacted mosquito collections, we estimated Pearson’s correlations (Sokal and Rohlf 1994) between times series for each of the six weather variables and the total number of mosquitoes, separated by sex, including all species, for each trap type and lure combination. We repeated this procedure for *Ae. sierrensis* samples, and in both cases selected the temporal scale for each weather variable based on the highest correlations found. After selecting the best temporal scale for each climatic variable, we proceeded to fit Poisson generalized linear mixed models (P-GLMMs) to mosquito abundance counts as a function of the following fixed factors: trap type, including rainfall and temperature; and the sampling location was treated as a random factor. This modeling strategy was selected to make an inference independent of the specific sampling locations of this study (Chaves 2010), and to account for the count nature of the collected data using a Poisson distribution (Bolker et al. 2009). Fixed factor significance was then tested using likelihood ratio tests between the full model, i.e., a model including the two weather variables and trap type, with simplified versions that removed one fixed factor at a time (Faraway 2006). For parameter inference, we performed a likelihood profile for each fixed factor that was then used to estimate the 95% confidence intervals (Bolker et al. 2009). For the models, we standardized rainfall and temperature data by removing their mean values and dividing the values by the time series standard deviation to ease the interpretation of the intercept parameters as the mean values of mosquitoes by trap type (Faraway 2004). All maps and analyses were made using the R language for statistical computing version 3.6.1 (R Core Team 2019).

## Results

During the 12 wk of this trial we collected a total of 684 mosquitoes. Besides *Ae. sierrensis* (32.9% of total catch), we also captured *Culiseta incidens* (Thomson) (41.5%), *Culex pipiens* L. (13.5%), *Culex tarsalis* Coquillet (10.5%), *Culex erythrothorax* Dyar (0.7%), *Culiseta inornata* (Williston) (0.6%), and *Aedes vexans* (Meigen) (0.2%) (Table 1). The BGS (CO_2_ and lure) traps captured 62% (*n* = 422) of all mosquitoes collected in the study, followed by the MM at 31% (*n* = 213), and both the BGS and BG Bowl contributed 3.5% (*n* = 24) each (Table 1). Mosquito samples collected were adult females for all species, except for *Ae. sierrensis* and *Cx. pipiens* for which male specimens were also collected (Table 1). The only trap which collected all seven mosquito species during this study was the BGS with CO_2_ and lure (Table 1). The sampling effort was slightly heterogeneous; collections at Downington started 1 wk after the two other sites, and on week 10 the BGS trap with lure at Allen Park failed.

**Table 1.** Mosquito species collected by trap type and bait, location, and sex (F = females, M = males) from suburban Salt Lake City, UT

Trap type	Location	*Culex pipiens*		*Culex tarsalis*	*Culex erythrothorax*	*Aedes sierrensis*		*Aedes vexans*	*Culiseta inornata*	*Culiseta incidens*
		F	M	F	F	F	M	F	F	F
Mosquito Magnet	Jones	4	1	11	0	1	14	0	1	18
	Downington	0	0	0	0	23	61	0	0	23
	Allen Park	2	0	9	0	17	19	0	0	9
BG Bowl + Lure	Jones	1	1	0	0	0	1	0	0	0
	Downington	8	0	1	1	1	0	0	0	0
	Allen Park	5	2	0	0	1	0	0	0	2
BGS + CO_2_ + Lure	Jones	5	0	5	0	5	18	1	2	24
	Downington	13	2	20	4	9	3	0	0	34
	Allen Park	35	3	23	0	27	24	0	0	165
BG + Lure	Jones	2	0	2	0	0	0	0	0	4
	Downington	0	0	0	0	1	0	0	1	4
	Allen Park	3	5	1	0	0	0	0	0	1

Temporal patterns of mosquito abundance show the overall mosquito community (Fig. 1D) followed rainfall pulses, increasing abundance when rainfall was low or absent (Fig. 1B). The MM traps were the only traps collecting relatively high numbers of males (Fig. 1D). The highest number of mosquitoes throughout the study period were collected by BGS (CO_2_ and lure) traps, followed by MM traps (Fig. 1D). Meanwhile, *Ae. sierrensis* (Fig. 1F) was proportionally more abundant during the first 6 wk of the study, when temperatures were below 26°C (Fig. 1C). The BGS (CO_2_ and lure) traps captured the largest share of mosquitoes from all species, followed by MM traps, which outperformed the BGS and BG Bowl traps with lure in the total number of mosquitoes captured (Fig. 1E). Females of *Ae. sierrensis* had similar numbers in the BGS (CO_2_ and lure) and the MM traps (Fig. 1G), the number of males was larger in the MM traps (Fig. 1H), a clear pattern that can also be observed temporally (Fig. 1E).

Correlation analyses, based on Pearson’s *r* coefficients, showed that 2-d rainfall and temperature had the highest correlation with female and male mosquito abundance. All the *r* estimates for 2-d variables were above (or below) + (−) 0.7, so these weather variables were used to fit the P-GLMMs. The maximum likelihood ratio tests showed that trap type, rainfall, and temperature had significant effects (*P* < 0.05), explaining differences in the number of male and female mosquito catches for all species, including *Ae. sierrensis* (Table 2). In all cases, rainfall and temperature had a negative effect, reducing the number of mosquitoes caught independently of the trap type, as indicated by estimates below one in Table 3. Briefly, in P-GLMMs the coefficients are not additive, but multiplicative, meaning that mosquito abundance estimates by trap type (also presented in Table 3) are multiplied by the rainfall and/or temperature estimates each time these weather variables are one unit above their average. Results from the P-GLMM (Table 3) confirm that effectively BGS (CO_2_ and lure) traps caught the largest number of mosquitoes per sampling period, and showed that female *Ae. sierrensis* numbers are similar in BGS (CO_2_ and lure) and MM traps; though MM traps captured slightly more male mosquitoes than any other of the deployed traps (Table 3). Also, it is important to note that Table 3 shows abundance estimates where the impact of rainfall and temperature has been removed. For example, without that consideration, the average number (±SD) of adult female *Ae. sierrensis* would have been 1.17 ± 2.93 in BGS (CO_2_ and lure) traps and 1.17 ± 2.66 in MM traps.

**Table 2.** Maximum likelihood ratio tests (LRT) for the significance of trap type, rainfall, and temperature on Poisson generalized linear mixed effects models for the abundance of all mosquito species, and *Aedes sierrensis*, separated by sex, across suburban habitats in Salt Lake City, UT

Species	Model	df	AIC	LRT	*P*(χ ^2^)
All species	Full model		**1093.2**		
Females	Trap type	3	1669	581.76	<2.20E-16*
	Rainfall	1	1112.6	21.42	<3.68E-06*
	Temperature	1	1204.3	113.13	<2.20E-16*
All species	Full model		**423.53**		
Males	Trap type	3	576.04	158.51	<2.20E-16*
	Rainfall	1	567.88	146.34	<2.20E-16*
	Temperature	1	600.14	178.61	<2.20E-16*
*Ae. sierrensis*	Full model		**240.75**		
Females	Trap type	3	326.44	91.695	<2.20E-16*
	Rainfall	1	271.05	32.304	<1.32E-08*
	Temperature	1	325.34	86.594	<2.20E-16*
*Ae. sierrensis*	Full model		**313.06**		
Males	Trap type	3	507.96	200.91	<2.20E-16*
	Rainfall	1	489.51	178.45	<2.20E-16*
	Temperature	1	540.01	228.95	<2.20E-16*

In the table, rows indicating ‘full model’ show data for models that included trap type, rainfall, and temperature as covariates. Rows indicating ‘trap type’, ‘rainfall’, and ‘temperature’ show results for the LRTs between the full model and models where that variable was removed. AIC stands for Akaike information criterion, a model selection metric that selects best models by minimizing the trade-off between model likelihood and parameter number (Kuhn and Johnson 2013). In the table, best models are **bolded**.

*Statistically significant (*P* < 0.05).

**Table 3.** Parameter estimates and 95% confidence intervals for Poisson generalized linear mixed effects models explaining the abundance of all mosquito species, and *Aedes sierrensis*, separated by sex, across suburban habitats in Salt Lake City, UT

Species	Parameter	Estimate	95% CI Lower limit	95% CI Upper limit
All species	Mosquito Magnet	2.593	1.117	5.961
Females	BG Bowl + Lure	0.461	0.282	0.717
	BGS + CO_2_ + Lure	8.174	6.668	10.094
	BGS + Lure	0.432	0.258	0.684
	Rainfall	0.787	0.710	0.871
	Temperature	0.524	0.463	0.591
	Location SD	0.506		
All species	Mosquito Magnet	1.191	0.603	2.279
Males	BG Bowl + Lure	0.050	0.015	0.131
	BGS + CO_2_ + Lure	0.627	0.442	0.962
	BGS + Lure	0.063	0.022	0.154
	Rainfall	0.261	0.197	0.337
	Temperature	0.175	0.124	0.238
	Location SD	0.362		
*Ae. sierrensis*	Mosquito Magnet	0.413	0.084	1.823
Females	BG Bowl + Lure	0.020	0.003	0.279
	BGS + CO_2_ + Lure	0.413	0.268	2.721
	BGS + Lure	0.010	0.001	0.200
	Rainfall	0.451	0.333	0.600
	Temperature	0.177	0.107	0.273
	Location SD	0.875		
*Ae. sierrensis*	Mosquito Magnet	0.692	0.307	1.489
Males	BG Bowl + Lure	0.0074	0.0004	0.0330
	BGS + CO_2_ + Lure	0.331	0.230	0.469
	BGS + Lure	0.000	0.000	0.000
	Rainfall	0.192	0.138	0.258
	Temperature	0.090	0.054	0.139
	Location SD	0.419		

Models were a function of trap type, temperature, and rainfall. Location SD is the parameter estimate for the random effect of the study locations.

## Discussion

Our data shows BGS with CO_2_ and lure traps were the most effective at catching mosquitoes, both in terms of abundance and species richness, within wooded suburban environments in Salt Lake City, Utah. The BGS (CO_2_ and lure) samples included *Ae. sierrensis* and all the other collected species, thus outperforming the other traps evaluated in this trial, some of which did not collect all of mosquito species. This is an important point because many of the other collected species are medically important and of public health significance. For example, *Cx. pipiens* and *Cx. tarsalis* are proven vectors of WNV in North America (Reisen et al. 2006a, Ruiz et al. 2010, Shand et al. 2016), while *Ae. vexans*, *Cx. erythrothorax*, *Cs. inornata*, and *Cs. incidens,* are species vectorially competent to transmit the virus (Turell et al. 2001, Goddard et al. 2002, Turell et al. 2005, Reisen et al. 2006b). From a veterinary perspective, *Ae. vexans* is also an important vector of dog heartworm (Bemrick and Sandholm 1966, Hendrix et al. 1980), while *D. immitis* infections have also been detected in *Cx. pipiens*, *Cx. tarsalis*, *Cx. erythrothorax, Cs. inornata*, and *Cs. incidens* (Huang et al. 2013).

In regards to *Ae. sierrensis*, our data is consistent with ecological patterns observed in other regions of North America, where adult mosquito abundance mainly shows a unimodal abundance peak (Woodward et al. 2003, Thiemann et al. 2017), a pattern suggested to reflect the univoltine biology of *Ae. sierrensis* (Hawley 1985). It should be noted that since *Ae. sierrensis* primarily overwinters as larvae, and juvenile development may be slow in the early spring/summer, additional synchronous broods may be observed following favorable precipitation conditions (Carpenter and LaCasse 1955, Farajollahi and Price 2013). Hatching of eggs occurs during initial fall or winter rains to allow for larval overwintering; however, spring rains may hatch additional eggs that were previously not flooded (Baerg 1965). Hence, overwintering may occur in the larval stage or in the egg stage if the tree holes have not been flooded (Judson et al. 1966).

During our investigations, *Ae. sierrensis* was more abundant when temperatures were lower during the section of spring and summer, further corroborating the larger unimodal peak generally observed in univoltine mosquito species. Additionally, *Ae. sierrensis* mosquito collections were also very sensitive to environmental conditions (temperature and precipitation) during our collection periods, a common pattern across several mosquito species and adult sampling methods (Yang et al. 2008, Barrera et al. 2011, Chaves et al. 2013, Chaves 2016). Interestingly, the negative impact of rainfall was immediate, suggesting rainfall more likely interfered with adult mosquito host seeking and flight activity, a condition necessary for mosquitoes to approach all the traps we tested (McDermott and Mullens 2018). This effect had a greater impact than changes related to adult mosquito recruitment from tree holes, which would result in rainfall impacting abundance over a longer time scale (Washburn and Anderson 1993, Washburn 1995). This latter observation should be tested in future studies that will span multiple seasons.

In our study, BGS (CO_2_ and lure) traps had a similar performance to MM traps in collecting female *Ae. sierrensis* adults; however, MM traps outperformed BGS (CO_2_ and lure) traps for collecting male *Ae. sierrensis* adults. This could be attributed to greater plumes of CO_2_ that are generated by MM traps and the larger size of those traps, which may be more attractive as swarm markers to male *Ae. sierrensis*. Nevertheless, because only female mosquitoes are a biting nuisance and of animal/public health importance, the comparable catch counts between the two trap types would operationally still favor the utility of the BGS (CO_2_ and lure) traps within SLCMAD’s surveillance program. These traps are also comparatively easier to operate than MM traps, both in transport and also in setup. The BGS (CO_2_ and lure) are much lighter (1.7 kg) as compared to the MM traps (15 kg) that we used during our investigations. The BGS (CO_2_ and lure) traps are also used with small compressed paintball-style CO_2_ tanks that weigh only about a kilogram, whereas an average propane tank used with MM traps is between 7 and 9 kg filled. Additionally, BGS traps allow the usage of different sources of CO_2_ (various CO_2_ tank sizes or dry ice), and are not strictly limited to an exclusive larger propane tank as utilized by the MM traps. The smaller size of the BGS traps and their collapsibility also allow for easier transport via vehicles into the field (many more can be deployed and stored), in addition to allowing more options for trap placement within residential backyards. The smaller size of the BGS traps also allow placement within more cryptic and hidden habitats, which would further reduce vandalism and theft in the field. Lastly, cost and part replacement should also be considered, as the BGS traps are more affordable (~$180 USD) and easier to maintain than the MM traps (~$330 USD). The BG Bowl trap and BGS with lure only did not perform as well as the other two trap types. This could be because *Ae. sierrensis* may not be as attracted to the BG lure as other container-inhabiting *Aedes* species. Future studies should incorporate other attractants in BGS traps, such as octenol, to test efficacy against *Ae. sierrensis*. All of these factors should be considered when selecting effective surveillance tools within operational programs.

In conclusion, the BGS (CO_2_ and lure) traps proved to be an effective and operationally feasible surveillance tool for *Ae. sierrensis* in suburban habitats of western United States. Additionally, the traps also proved effective for collection of other medically and veterinary important mosquitoes, such as *Cx. pipiens* and *Cx. tarsalis*. Efficacy, economics, and operational ease of use are all important factors which have positively contributed to selection of the BGS (CO_2_ and lure) traps as a primary surveillance (and control) tool against *Ae. sierrensis* within SLCMAD’s tree hole mosquito program. In particular, since *Ae. sierrensis* adults are weak fliers and do not disperse far from larval tree hole habitats, removal trapping using BGS (CO_2_ and lure) traps may be a viable control method for focal populations within private residences. Modern mosquito surveillance and control programs utilizing integrated mosquito management techniques must rely on providing not only public health benefits, but also enhancement to quality of life. Effective reduction of biting adult mosquitoes would address both of these concerns and lead to responsible public health stewardship for the benefit of the general public and associated pets.
