# Erratum to “Trap Comparison for Surveillance of the Western Tree Hole Mosquito, *Aedes sierrensis* (Diptera: Culicidae)”

**DOI:** 10.1093/jisesa/ieaa007

**Published:** 2020-02-29

Correction of “Chaves L. F., N. Reissen, G. S. White, S. Gordon, and A. Faraj. 2020. Trap Comparison for Surveillance of the Western Tree Hole Mosquito, *Aedes sierrensis* (Diptera: Culicidae) J. Insect Sci.”

DOI: 10.1093/jisesa/iez131

During the processing of this article, a miscommunication between the author and the publishing team resulted in the title appearing incorrectly in the initial published version online. The title has been corrected.

